# Female-Biased Symbionts and Tomato Yellow Leaf Curl Virus Infections in *Bemisia tabaci*

**DOI:** 10.1371/journal.pone.0084538

**Published:** 2014-01-22

**Authors:** Huifang Guo, Yufeng Qu, Xiangdong Liu, Wanfang Zhong, Jichao Fang

**Affiliations:** 1 Institute of Plant Protection, Jiangsu Academy of Agricultural Sciences, Nanjing, People's Republic of China; 2 Department of Entomology, Nanjing Agricultural University, Nanjing, People's Republic of China; Institute of Infectious Disease and Molecular Medicine, South Africa

## Abstract

The female-biased infection of facultative symbionts has been found in *Bemisia tabaci*; however, whether there are any differences in tomato yellow leaf curl virus (TYLCV) and obligate symbiont infection rates between females and males is unknown. Determining whether such differences exist would be very important for understanding the spread of the plant virus and of the symbionts. We compared both symbiont infection types, including obligate and facultative symbionts, and the rates of TYLCV infection in both sexes in five field populations from Jiangsu Province, China. The obligate symbiont *Portiera aleyrodidarum* was not found in every whitefly tested. In all tested populations, more females than males were found to harbor *P. aleyrodidarum*; and more females than males also harbored *Hamiltonella defense*, the most common facultative symbiont as well as *Cardinium*. In addition to female-biased symbiont infections, there were also female-biased TYLCV infections, and the infection frequencies of this plant virus in females were higher than those in males. Taken together, these results suggested that both the female-biased symbiont infections and female-biased TYLCV infections promoted the rapid spread of TYLCV in China.

## Introduction

Insects commonly harbor a variety of endosymbionts, including obligate symbionts that are essential for the host's survival and development and facultative symbionts that are not essential but may play important roles in the host's ecology and evolution. The relationships between symbionts and insects have been studied in many insects, especially in aphids. The symbiont community in aphids has been found to be highly diverse and dynamic, with different symbionts being identified among host species and among individual hosts within species [1]. There are diverse beneficial effects conferred by the symbionts of aphids, including protection from natural enemies, heat tolerance, insecticidal susceptibility, and improvements in reproduction [1], [2], [3].

*Bemisia tabaci* (Hemiptera: Aleyrodidae) is a worldwide insect pest that threatens many crops and is a major problem not only as a plant pest but also as a vector of plant viruses [4], [5]. Similar to other sap-sucking insects, such as aphids, *B. tabaci* also harbors obligate and facultative symbionts. *Portiera aleyrodidarum* is an obligate symbiont in *B. tabaci*, and *Arsenophonus*, *Hamiltonella*, *Wolbachia*, *Cardinium*, *Fritschea* and *Rickettsia* are facultative symbionts that have been found in *B. tabaci* [6], [7], [8], [9], [10]. Studies on facultative symbiont diversity among different biotypes of *B. tabaci* have indicated that some facultative symbionts were biotype-dependent. In Israel, only the B biotype harbors *Hamiltonella*, only the Q biotype harbors *Wolbachia* and *Arsenophonus*, and both biotypes harbor *Rickettsia* [7]. In the southwest Indian Ocean, a clear association between facultative symbiotypes and biotypes of *B. tabaci* was also identified. The B biotype, which is an invasive biotype in the region, harbors *Hamiltonella*, the Ms biotype, which is an indigenous biotype, harbors *Cardinium* and *Arsenophonus*, and both of the biotypes harbor *Rickettsia* [11]. Aside from the differences in symbiont species, differences in symbiont combinations among *B. tabaci* have also been described among biotypes, which suggested that the symbionts might function in the differentiation and invasion of biotypes [7], [11]. In addition to the variation in symbiont infections, genetic diversity was also found in symbionts among different biotypes or populations. Among the worldwide populations of the B and Q biotypes of *B. tabaci*, two strains of *Wolbachia*, four strains of *Arseophonus* and two strains of *Cardinium* have been found [12]. In addition to the differences in the facultative symbiont communities among different geographical populations or biotypes, a variation in the facultative symbiont communities between female and male individuals of *B. tabaci* has also been identified, and the infection frequencies of *Hamiltonella* in females was higher than those in males in 6 studied field populations [13]. In 5 experimental subpopulations of whiteflies, several facultative symbiont infections, including *Hamiltonella*, *Rickettsia* and *Cardinium* infections, were female-biased infections [14]. Whether female-biased facultative symbiont infections are common in the natural population is still unknown. Aside from *Hamiltonella*, whether there are sexual differences among the other facultative symbionts in field populations of *B. tabaci* also remains unclear.

Previous survey has indicated that obligate symbionts infected every individual of all the tested *B. tabaci* populations [15]. Answering the questions regarding whether there are individuals that are not infected with obligate symbionts and whether there are sexual differences among obligate symbiont infections would be beneficial to understanding the role of symbionts and to provide possible methods for the efficient control of the whitefly population through the manipulation of their symbionts.

Tomato yellow leaf curl virus (TYLCV) (family Geminiviridae, genus Begomovirus) is the most important plant virus that is transmitted by *B. tabaci* [16]. The transmission of TYLCV by whiteflies is affected by many factors associated with the vector, including sex, biotype and endosymbionts. It has been reported that the transmission of TYLCV was related to the sex of the whiteflies and that viruliferous females could transmit TYLCV to males but not to females, and similarly, viruliferous males could transmit the virus only to females, suggesting that TYLCV transmission exclusively occurs among whiteflies of different sexes [17]. The sex of *B. tabaci* also affected the efficiency of TYLCV transmission, with female whiteflies observed to transmit the virus more efficiently than males [18]. However, whether the infection frequencies of TYLCV in females are higher than those in males is still poorly understood.

In the present study, we compared the symbiont communities, including obligate and facultative symbionts, among female and male individuals of *B. tabaci* from 5 field populations. At the same time, we compared the TYLCV infection frequencies between the two sexes. Based on these data, we analyzed the association between symbiont populations or TYLCV infection rates and sex bias.

## Materials and Methods

### Ethics statement

The whiteflies used in our studies were collected from the Experimental Station of Jiangsu Academy of Agricultural Sciences. No specific permissions were required for these locations. We confirm that the locations are not privately-owned or protected in any way, and our field studies do not involve endangered or protected species.

### Whitefly collection

*Bemisia tabaci* adults were collected from August to September 2011 from cotton plants in Fengxian Jiangsu province, and from cotton, sweet potato, tomato and cucumber plants in Nanjing, Jiangsu province, China. The sexes of the collected adults were identified, and the whiteflies from both sexes were separately stored at −20°C for DNA extraction.

### DNA extraction

Each field-collected whitefly was ground individually with a sterilized toothpick for 1 min in 20 µL extraction buffer (50 mM Tris–HCl pH 8, 20 mM NaCl, 1 mM EDTA, and 1% SDS). Then, 1 µL 20 mg/ml proteinase K (Sigma) was added to the extract and the mixture was centrifuged. The lysate was then incubated at 60°C for 1 h, 1 µL 20 mg/mL proteinase K (Sigma) was again added to the liquid, and the lysate continue to be incubated at 60°C for 2 h, followed by the addition of 178 µL ddH_2_O and further incubation at 100°C for 5 min. Then, 100 µL extract was removed to new Eppendorf tube, 200 µL alcohol was then added to the new tube and the mixture was stored at −20°C overnight. The next day, the extract was centrifuged, the suspension was poured out, 20 µL ddH_2_O was added to the DNA, and the DNA was stored at −20°C until use.

### Biotype Identification

Biotypes were identified based on the amplification of the mitochondrial Cytochrome Oxidase (mtCOI) gene. The primers that were used for amplifying the gene are shown in Table 1. The 25 µL PCR reaction system contained the following: 2 mM Mg^2+^, 200 mM dNTP, 1×PCR buffer, 0.5 U DNA Taq enzyme, and 0.4 µM each primer. The PCR program was as follows: denaturation at 94°C for 5 min, followed by 35 cycles at 94°C for 90 s, 50°C for 90 s and 72°C for 1 min, and a final extension step at 72°C for 10 min. The PCR product was sequenced to identify the biotype. The bands which were the PCR products visualized using 2% agarose gel were eluted and cloned into the pGEM T-Easy plasmid vector (Promega). The vectors were transformed into *E. coli* and sequenced. The sequences were then compared with the known sequences databases of the Natural Center for Biotechnology Information (NCBI) to identify the biotype.

**Table 1:** Nucleotide sequence of the primers that were used in the present study.

Target organism	Target gene	Primer name	Primer sequences (5′-3′)	Product size(bp)	References
*B. tabaci*	Mt COI	C1-J-2195	TTGATTTTTTGGTCATCCAGAAGT	800	[18]
		L2-N-3014	TCCAATGCACTAATCTGCCATATTA		
*P. aleyrodidarum*	16SrDNA	28F	TGCAAGTCGAG CGGCATCAT	1000	[19]
		1098R	AAAGTTCCCGCCTTATGCGT		
*H. defense*	16SrDNA	92F	TGAGTAAAGTCTGGGAATCTGG	1250	[19]
		1343R	CCCGGGAACGTATTCACCGTAG		
*C. hertigii*	16SrDNA	Ch-F	TACTGTAAGAATAAGCACCGGC	400	[20]
		Ch-R	GTGGATCACTTAACGCTTTCG		
*Rickettsia*	16SrDNA	Rb-F	GCTCAGAACGAACGCTATC	900	[8]
		Rb-R	GAAGGAAAGCATCTCTGC		
*Wolbachia*	wsp	81F	TGGTCCAATAAGTGATGAAGAAAC	600	[21]
		691R	AAAAATTAAACGCTACTCCA		
*Arsenophonus*	23srDNA	Ars23-1	CGTTTGATGAATTCATAGTCAAA	600	[22]
		Ars23-2	GGTCCTCCAGTTAGTGTTACCCAAC		
*Fritschea*	23srDNA	U23F	GATGCCTTGGCATTGATAGGCGATGAAGGA	600	[23]
		23SIGR	TGGCTCATCATGCAAAAGGCA		

### Endosymbiont detection

The symbiont communities and infection frequencies among female and male whiteflies within the population were compared. Sixty field-collected adults of each sex were used to detect the presence of obligate symbionts and facultative symbionts on each individual. Mt COI gene amplifications of all the samples were completed prior to symbiont detection to test the quality of the DNA extraction, and the results showed that all of the DNA extracts used for symbiont detection were of high quality. The 16S or 23SrDNA gene fragments were amplified to determine the presence of symbionts, and all the primers that were used to detect the obligate symbiont *P. aleyrodidarum* and the facultative symbionts *Hamiltonella*, *Rickettsia*, *Wolbachia*, *Arsenophonus*, *Cardinium* and *Fritschea* are listed in Table 1. The DNA quality of all individuals for symbiont detection was confirmed by the successful cloning of the mt COI gene. The PCR reaction system and program used for each symbiont were the same as those described in the references [8], [19]–[24].

To confirm that the amplified bands were actually from the desired symbionts, bands for each symbiont were randomly selected, sequenced, and compared with the known sequences databases from NCBI.

### Tomato yellow leaf curl virus detection

The tomato yellow leaf curl virus infection frequencies in female and male whiteflies were compared. The DNA samples used for symbiont detection were also used for TYLCV detection. As with symbiont detection, 60 adults for each sex were used to detect the presence of TYLCV in each individual. The specific primers TV_P833 (5′-GGTCTACAC GCTTACGCCTTATT-3′) and PATY_R (5′-TTCCATCCGAACATTCAGGCAGC-3′) were used to amplify the TYLCV DNA, and the PCR products were 800 bp. The 10 µL PCR reaction system included the following: 5 µL mix (100 mM KCl, 20 mM Tris-HCl, 3 mM MgCl_2_, 400 µM dNTPs), 0.2 µL Taq enzyme (5 U/µL), 0.5 µL TV_P833 primer, 0.5 µL PATY_R primer, 3.2 µL H_2_O, and 0.6 µL DNA. The PCR program was as follows: denaturation at 94°C for 5 min, followed by 35 cycles at 94°C for 45 s, 56°C for 45 s and 72°C for 1 min, and a final extension step at 72°C for 10 min. The PCR product was sequenced to identify the virus species.

### Statistical analyses

The symbiont and TYLCV infection frequencies between females and males were compared by a Pearson non-parametric test using the χ^2^ distribution. SPSS software 11.0 was used for statistical analyses.

## Results

### 1. Differences in obligate symbiont infection rates in female and male *B. tabaci*

Five field populations of *B. tabaci* were collected in Jiangsu province, China to compare the symbiont infection rates between females and males. Before symbiont detection, biotype identification was conducted based on mtCOI sequences, and the results indicated that all the individuals from the single population collected in Fengxian, Jiangsu province belonged to the B biotype, and all of the populations collected in Nanjing, Jiangsu province belonged to the Q biotype.

**Figure 1:**
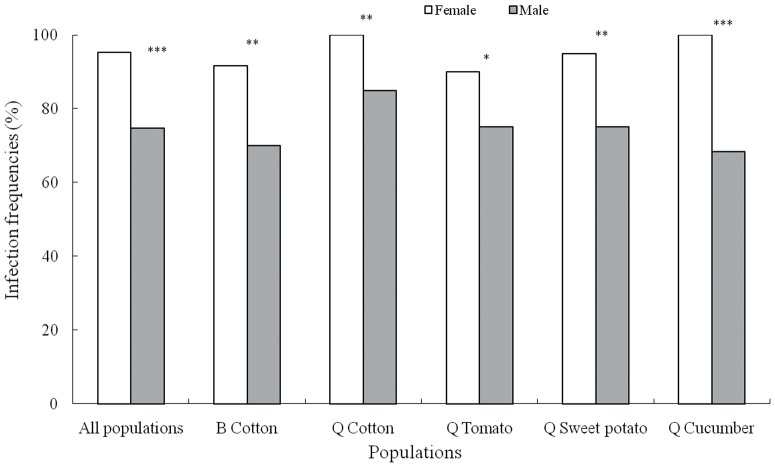
Infection frequencies of the obligate symbiont in female and male *B. tabaci*. Columns indicate a female-biased obligate symbiont, *P. aleyrodidarum* infection in the 5 populations of whiteflies; and the infection rate in females in each population was all significantly higher than that in males. *P<0.05, **P<0.01, and ***P<0.0001.

The obligate symbiont communities in both female and male adults in the one B biotype and four Q biotype populations were compared (Fig. 1). The obligate symbiont *P. aleyrodidarum* was not found in every tested individual whitefly in any of the 5 populations examined, and a female-biased infection frequency of the obligate symbiont in *B. tabaci* was observed. The overall infection rate in females from the 5 populations was significantly higher than that in males (95.3% and 74.7%, respectively, χ^2^_1_ = 51.413, *P*<0.0001). Whether the population was collected from cotton, tomato, cucumber or sweet potato, the infection frequencies in females were always significantly higher than those in males. In the populations of the B and Q biotypes collected from cotton, 100% and 91.7% of females, respectively, harbored the obligate symbiont, whereas only 85% and 70% of males, respectively, were found to harbor *P. aleyrodidarum* (B: χ^2^_1_ = 9.09, *P* = 0.0023, Q: χ^2^_1_ = 9.7297, *P* = 0.0014). In populations of the Q biotype collected from tomato, sweet potato and cucumber, the infection rates in females were all higher than 90%, and those in males were all lower than 75% (tomato: χ^2^_1_ = 4.6753, *P* = 0.0264; sweet potato: χ^2^_1_ = 9.4118, *P* = 0.0019; and cucumber: χ^2^_1_ = 22.5743, *P*<0.0001).

### 2. Differences in facultative symbiont infection rates in female and male *B. tabaci*

All six known facultative symbionts in *B. tabaci* were tested, and four species of facultative symbionts, including *Hamiltonella*, *Rickttesia*, *Cardinium* and *Wolbachia*, were detected, whereas *Arsenophonus* and *Fritschea bemisiae* were not observed in any of the samples.

#### Hamiltonella infection

**Figure 2:**
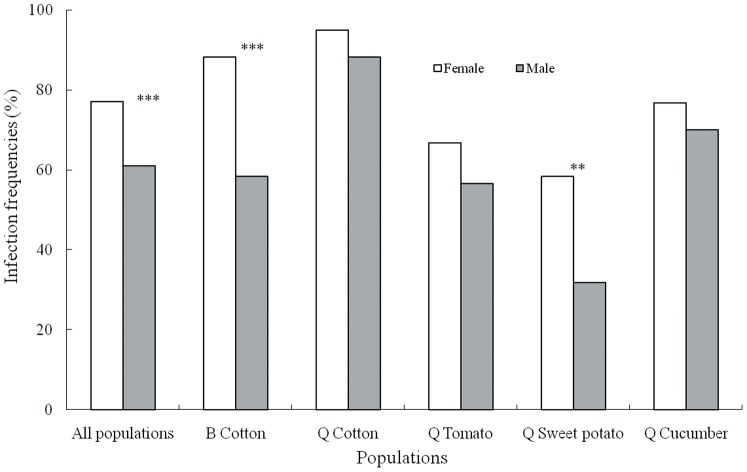
Infection frequencies of the facultative symbiont *Hamiltonella* in female and male *B. tabaci*. *Hamiltonella* was the predominant facultative symbiont identified in the 5 populations, and the overall infection frequency of *Hamiltonella* in females was significantly higher than that in males, whereas infection frequencies did not differ between females and males in every population; in two populations of the whiteflies, more females than males harbored *Hamiltonella*, whereas in the other three populations, the *Hamiltonella* infection frequencies of females and males were at similar levels.

*H. defense* was the predominant facultative symbiont identified in the 5 populations (Fig. 2). Seventy-seven percent of females and 61% of males harbored *Hamiltonella*, and the infection frequency was significantly different between the two sexes (χ^2^_1_ = 17.9523, *P*<0.0001). *Hamiltonella* infection frequencies did not differ between females and males in every population; in populations of the B biotype collected from cotton and in populations of the Q biotype collected from sweet potato, more females than males harbored *Hamiltonella* (B cotton: χ^2^_1_ = 13.8068, *P* = 0.0002; Q sweet potato: χ^2^_1_ = 8.6195, *P* = 0.0028), whereas in populations of the Q biotype collected from cotton, tomato and cucumber, the *Hamiltonella* infection frequencies of females and males were at similar levels (Q cotton: χ^2^_1_ = 1.7455, *P* = 0.1612; Q tomato: χ^2^_1_ = 1.1291, *P* = 0.174; Q cucumber: χ^2^_1_ = 0.6818, *P* = 0.2681).

#### *Cardinium*, *Wolbachia* and *Rickettsia* infections

*Cardinium*, *Wolbachia* and *Rickettsia* infections were all observed at low frequencies (Table 2). As was observed with the *Hamiltonella* infection, the overall infection frequencies of *Cardinium* and *Rickettsia* were also significantly different between females and males, but the sex-biases of the infections were different; more females (20.7%) than males (12.7%) were infected with *Cardinium* (χ^2^_1_ = 6.912, *P* = 0.0057), whereas more males (8.7%) than females (3.3%) were infected with *Rickettsia* (χ^2^_1_ = 7.565, *P* = 0.0045). Neither *Cardinium* nor *Rickettsia* were differently distributed among female and male whiteflies in different populations, and only the population of the Q biotype collected from cucumber showed a *Cardinium* infection difference between females and males (25% and 6.7%, respectively, χ^2^_1_ = 7.5664, *P* = 0.0055), while only the population of the Q biotype collected from tomato showed a difference in *Rickettsia* infection between females and males (0% and 31.7%, respectively, χ^2^_1_ = 22.5743, *P*<0.0001). No *Wolbachia* infection associated with sex was found in any of the populations.

**Table 2:** Infection frequencies of facultative symbionts in female and male *B. tabaci*.

Biotype	Host plant	Sex	Infection frequency (%)		
			*Cardinium*	*Rickettsia*	*Wolbachia*
B	Cotton	F	20	13.3	8.3
		M	21.7	8.3	18.3
Q	Cotton	F	16.7	0	0
		M	11.7	0	0
	Tomato	F	16.7	0	0
		M	6.7	31.7***	0
	Sweet potato	F	25	0	0
		M	16.7	3.3	0
	Cucumber	F	25**	3.3	1.7
		M	6.7	0	0
Average		F	20.7**	3.3	2
		M	12.7	8.7**	3.7

Note:

*P*<0.01.

*P*<0.0001.

### 3. Multiple infections of facultative symbionts in female and male *B. tabaci*

Multiple infections of facultative symbionts were also compared in female and male *B. tabaci* (Table 3). Although most of the individuals harbored only one species of facultative symbiont, seven types of multiple infections, including two types of triple infections and five types of double infections, were also found, and they were all observed at low frequencies. There were differences observed in multiple infection frequencies between females and males. Among the multiple infections, the HR (*Hamiltonella* and *Rickettsia*) and HC (*Hamiltonella* and *Cardinium*) infection frequencies differed between females and males. The infection frequency of HR in females (2%) was significantly lower than that in males (6.7%) (χ^2^_1_ = 7.88, *P* = 0.004), whereas the infection frequency of HC in females (17%) was significantly higher than that in males (6%) (χ^2^_1_ = 17.833, *P*<0.0001). The infection frequencies in females and males did not differ for the other five types of multiple infections. HCW (*Hamiltonella*, *Cardinium* and *Wolbachia*) and HCR (*Hamiltonella*, *Cardinium* and *Rickettsia*) infections in females and males were both observed at the very low frequency of 0.3% (1/300). The HW (*Hamiltonella* and *Wolbachia*), CW (*Cardinium* and *Wolbachia*) and RW (*Rickettsia* and *Wolbachia*) infection frequencies were also not different between females and males. The population of females (17%) without any facultative symbionts was significantly lower than that of males (29.7%) (χ^2^_1_ = 13.453, *P*<0.0001).

**Table 3:** Multiple infection frequencies of facultative symbionts in female and male *B. tabaci*.

Biotype	Host plant	Sex	Infection frequency (%)							
			HCR	HCW	HW	HR	HC	CW	RW	N
B	Cotton	M	1.7	1.7	5	10	15	0	0	3.3
		F	0	1.7	10	6.7	10	5	0	26.7
Q	Cotton	M	0	0	0	0	16.7	0	0	5
		F	0	0	0	0	6.7	0	0	5
	Tomato	M	0	0	0	0	15	0	0	35
		F	0	0	0	26.7	1.7	0	0	35
	Sweet potato	M	0	0	0	0	15	0	0	28.3
		F	0	0	0	0	6.7	0	0	53.3
	Cucumber	M	0	0	0	0	23.3	0	1.7	13.3
		F	0	0	0	0	5	0	0	28.3
Average		M	0.3	0.3	1	2	17***	0	0.3	17
		F	0.3	0.3	2	6.7**	6	1	0	29.7***

Different letters represent different symbiont combinations: HCW, *Hamiltonella*, *Cardinium* and *Wolbachia*; HC, *Hamiltonella* and *Cardinium*; HR, *Hamiltonella* and *Rickettsia*; HW, *Hamiltonella* and *Wolbachia*; and CW, *Cardinium* and *Wolbachia*.

*P*<0.01,

*P*<0.0001.

### 4. Infection frequencies of TYLCV in female and male *B. tabaci*

**Figure 3:**
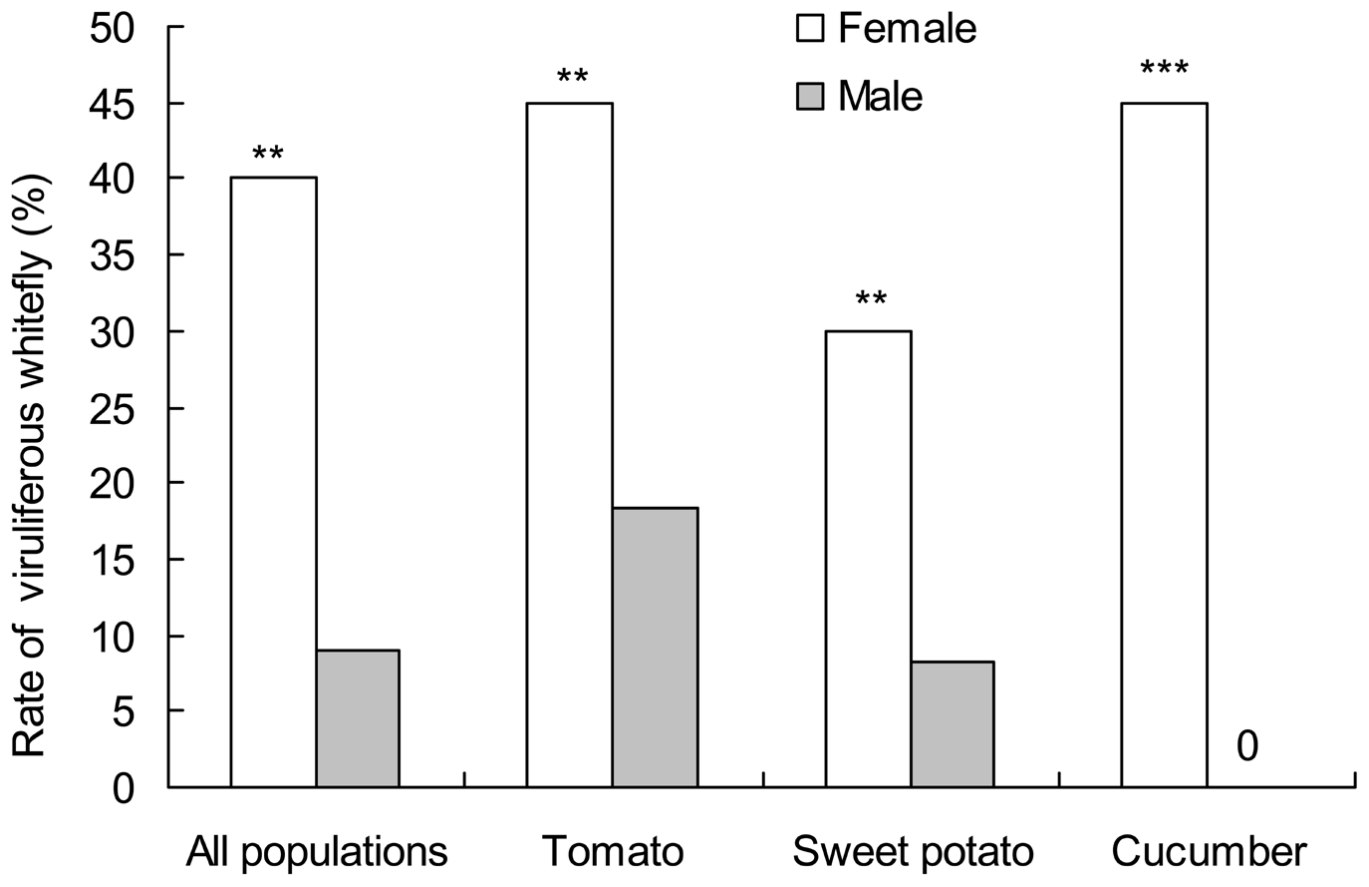
Rates of female and male viruliferous whiteflies. The TYLCV infection was sexual different, and the rate of viruliferous whiteflies in females was significantly higher than that in males.

Field populations of *B. tabaci* from tomato, cucumber and sweet potato were collected from the same location to compare the differences in TYLCV infection rates between females and males (Fig. 3). It was found that the three populations, the overall TYLCV infection frequency was 40% in female individuals, whereas it was only 8.9% in male individuals, and the rate of viruliferous whiteflies was significantly different between females and males (χ^2^_1_ = 47.166, *P*<0.0001). And in every population, the rates of viruliferous whiteflies in females were all significantly higher than that in males (Tomato: χ^2^_1_ = 9.859, *P* = 0.001; Sweet potato: χ^2^_1_ = 9.09, *P* = 0.002; Cucumber: χ^2^_1_ = 72, *P*<0.0001).

## Discussion

As an obligate symbiont in *B. tabaci*, *P. aleyrodidarum* benefits its host insect by providing nutrients that are lacking or limited in the diet, and all of the previous studies have indicated that *P. aleyrodidarum* was present in all individual whiteflies, as the obligate symbiont was thought to be required for host development and survival [13], [15], [25]. However, in our present study, this obligate symbiont was not detected in all of the tested female and male individuals of *B. tabaci*. In the five field populations collected in Fengxian and Nanjing, Jiangsu Province, China, the infection frequencies of obligate symbiont were not 100% in either females or males. There are several possible explanations for the absence of obligate symbiont in our study, one is that the populations of *B. tabaci* in our study is actually different from that in previous studies. Another is that the diagnostic detection method in our study is not sensitive enough as quantitative PCR to detect the lower symbiont titer in some individuals. In a natural population of the pea aphid, *Acyrthosiphon pisum*, the obligate symbiont *Buchnera aphidicola* was found to be present in nearly all individuals [26]. A facultative endosymbiotic γ-proteobacterium was found to compensate for the loss of the obligate symbiont, and aphids with the complete elimination of *Buchnera* could survive and reproduce after infection with the endosymbiont [27]. In our present study, most of the obligate symbiont-free *B. tabaci* harbored at least one facultative symbiont, and very few individuals had no symbiont. The presense of *P. aleyrodidarum* in immature stages of *B. tabaci*, and the adult whiteflies without this obligate symbiont should be further investigated.

Our present study also indicated that the obligate symbiont infection in *B. tabaci* has a female bias, and more males than females were found to be Portiera-free in all of the 5 field populations tested. As *B. tabaci* is a haplo-diploid species, diploid eggs produced by mated females will develop into females, and haploid eggs produced by unmated or mated females will develop into males [28]. It is reasonable to infer that, as females are the offspring of mated females, they have the opportunity to obtain symbionts from both the mother and the father, whereas because males are the offspring of unmated or mated females, their overall chances for obtaining symbionts are reduced, which may result in more females than males harboring the obligate symbiont.

In our present study, four of the six known facultative symbionts in *B. tabaci* were found in both B biotype and Q biotype whiteflies, including *Hamiltonella*, *Rickettsia*, *Cardinium* and *Wolbachia*. *Arsenophonus* and *Fritschea* were not observed. Among the four facultative symbionts, *Hamiltonella* was the most abundant symbiont in the B biotype, which is similar to what was observed in the B biotype from Croatia [29] and in the B and Q biotypes from China [13]. In the present study, *Cardinium* was less abundant, and *Wolbachia* and *Rickettsia* were rare symbionts. These results are in contrast to the facultative infection rates observed in the B biotype of *B. tabaci* from Israel, where *Rickettsia* was abundant, and *Cardinium* and *Fritschea* were not found [7]. In the B biotype from the southwest Indian Ocean, La Réunion, *Hamiltonella* and *Rickettsia* were all widely distributed [11]. In whiteflies from other locations in China, *Hamiltonella* and *Rickettsia* were both the predominant symbionts [13], [30]. The difference between our results and other reports from China may result from the differences in the sample collection sites or in change in symbiont frequencies over time. In the Q whiteflies in our present study, *Hamiltonella* was also the most abundant symbiont. This is consistent with previous findings in Q whiteflies in other reports from China. Gueguen et al [12] and Chiel et al [7] also found that *Hamiltonella* was present at a high rate in Q biotype whiteflies, whereas the abundance of *Arsenophonus*, *Rickettsia*, and *Wolbachia* was dependant on the locations of the Q biotype whiteflies. In our present study, the other three facultative symbionts were all observed at relatively low frequencies.

Female-biased *Hamiltonella* infection was also observed in the pooled data from the one B and four Q biotype field populations from Jiangsu province, China. However, the sexual difference varied with the populations, with female-biased *Hamiltonella* infections only found in two of the five populations: the B biotype population collected from cotton and the Q biotype population collected from sweet potato. In addition to the sexual differences in *Hamiltonella* infection frequencies, *Cardinium* and *Rickettsia* infection frequencies were also observed to be sex-dependent; more females than males harbored *Cardinium*, but more males than females harbored *Rickettsia*, although all infection frequencies were observed at low levels.

Multiple infections of symbionts in *B. tabaci* are common, and the combinations of symbionts are varied [7], [11]. In our present study, six types of multiple infections were detected in B biotype whiteflies, and three types were detected in Q biotype whiteflies. Two types of triple infections and five types of double infections were observed, and they were all observed at low frequencies. Sexual differences were also found in multiple infection frequencies, with HR and HC infection frequencies differing between females and males.

Symbiont-mediated function has been reported in many symbiont-host associations. For *Hamiltonella*, which was the most abundant facultative symbiont observed in our present study, previous reports showed that *Hamiltonella* was related to TYLCV transmission in *B. tabaci*, and the high infection rate of *Hamiltonella* in whitefly symbionts has been shown to promote the rapid spread of TYLCV in China [31], [32]. No other effect of *Hamiltonella* has been found in *B. tabaci*. In *A. pisum*, *Hamiltonella* has been shown to protect the host from being parasitized, and the protection capability varies among different symbiont strains [33]. In *A. pisum*, the infection of a facultative symbiont has been shown to compensate for the loss of the obligate symbiont [27]. It therefore may be possible for *Hamiltonella* to function in a similar role as the obligate symbiont in *B. tabaci*. *Rickettsia* in *B. tabaci* was found to function as a reproductive manipulator and mutualist, as it induced female bias and increased performance, and these properties have promoted the spread of *Rickettsia* across the southwestern United States [34]. Moreover, *Rickettsia* was also found to be associated with an increase in insecticide resistance [35]. However, the infection frequencies of *Rickettsia* were found to be very low in our present study, suggesting that its role is less important in the investigated populations.

Our present results showed that more females than males carried TYLCV in *B. tabaci*. Our present data and previous results have also demonstrated that more females than males harbor *Hamiltonella*. *Hamiltonella* has been found to be closely associated with the acquisition, retention and transmission efficiency of TYLCV [32]. The sex of *B. tabaci* was also found to affect the efficiency of TYLCV transmission, with female whiteflies observed to transmit the virus more efficiently than males [18]. Combined with the above, these results indicated that more females harboring *Hamiltonella* further promoted the expansion of TYLCV, and this could be a reasonable explanation for why TYLCV has spread so rapidly and has caused such heavy damage in the China. In future studies, it will be very important to explore methods for decreasing the infection frequency of *Hamiltonella* and, thus, inhibiting the spread of TYLCV.
